# Tryptophan intake, not always the more the better

**DOI:** 10.3389/fnut.2023.1140054

**Published:** 2023-04-11

**Authors:** Dongmei Hu, Junyi Liu, Wanlin Yu, Chuan Li, Lihua Huang, Wei Mao, Zhaoyu Lu

**Affiliations:** ^1^State Key Laboratory of Dampness Syndrome of Chinese Medicine, The Second Affiliated Hospital of Guangzhou University of Chinese Medicine, The Second Clinical College of Guangzhou University of Chinese Medicine, Guangzhou, China; ^2^Nephrology Department, The Second Affiliated Hospital of Guangzhou University of Chinese Medicine, Guangzhou, China

**Keywords:** tryptophan, kynurenine, indoxyl sulfate, aryl hydrocarbon receptor, chronic kidney disease

## Abstract

**Objectives:**

To investigate the effects of excessive tryptophan intake on the body and the effects of tryptophan metabolism-related aryl hydrocarbon receptor (AhR) pathway in healthy rats and chronic kidney disease rats, to study the adverse effects of excess tryptophan.

**Design:**

In Part I Experiment, the healthy rats were fed with diet containing 0.6, 1.2 and 1.8% tryptophan for 12 weeks. After the intervention, the blood and kidney tissues were collected. Serum creatinine and blood urea nitrogen were detected. Hematoxylin–eosin (H&E) staining was used to observe renal pathological changes. Enzyme-linked immunosorbent assay was used to detect serum kynurenic acid and AhR levels. The kidney levels of AhR, CyP1A1 and CyP1B1 were detected by western-blot. In Part II Experiment, the chronic kidney disease (CKD) model was induced by intra-gastric gavage with adenine for 4 weeks. Then the CKD rats were given tryptophan at a dose of 100 mg/kg or 500 mg/kg for eight weeks. Rat survival curve, renal function, renal tissue pathology and serum AhR were detected. Tryptophan-targeted ultra-high-performance liquid chromatography coupled with multiple reaction monitoring mass spectrometry (UHPLC-MRM-MS) was employed to quantitatively access the tryptophan-targeted metabolites in two parts experiments.

**Results:**

In part I experiment, high tryptophan diet can increase the level of blood urea nitrogen (BUN) in healthy rats and induce focal renal tubulointerstitial injury. Tryptophan-targeted analyzes showed that high tryptophan diet feeding can significantly increase the concentration of kynurenine and indole metabolites. The serum AhR level and kidney AhR, CyP1A1 and CyP1B1 were also significantly increased in high tryptophan diet rats. In part II experiment, high tryptophan intervention induced a significant increase in mortality, serum creatinine, urea nitrogen levels, and renal pathological damage in CKD rats. The levels of tryptophan-targeted metabolites, kynurenine, xanthurenate, picolinic acid, 5-hydroxyindole-3-acetic acid, indole-3-lactic acid, indoleacetate and indoxyl sulfate, showed an upward trend in the high-dose tryptophan group (Ade + Trp-H) compared with the adenine group. The serum AhR of Ade + Trp-H rats was significantly higher than those of adenine rats.

**Conclusion:**

Moderate tryptophan intake may be beneficial, but excessive tryptophan can lead to accumulation of kynurenine and indole metabolites, activate AhR pathway and induce kidney injury.

## 1. Introduction

Tryptophan (Trp) is an essential amino acid of aromatic family, which mainly derives from endogenous degradation of tissue proteins and dietary intake (1). Common natural food sources of TRP include soy, cashews, cocoa and whey products (2). It is not only an indispensable component in protein synthesis, but also plays an important role in maintaining tissue activity and function. Up to now, there have many studies on the effects of inadequate tryptophan intake on human disease, such as brain injury (3), cancer, neurodegenerative diseases (4), and autoimmune diseases (5).

Dietary tryptophan is an essential amino acid for intestinal mucosal cells, which participates in the regulation of intestinal immune system, microbiota, epithelial barrier and homeostasis (6). A review even indicated that excessive daily intake of tryptophan had protective effect on maintaining intestinal homeostasis (7). It seems like the more tryptophan intake is always better.

Tryptophan absorbed into body is metabolized in five pathways: kynurenine pathway (KP), serotonin pathway, gut microbial pathway and indol-3-ylpyrvic acid pathway and tryptamine pathway. Many studies focused on the tryptophan-serotonin pathway, indicating the protective effect of tryptophan supplementation. However, 95% of dietary tryptophan is metabolized through the kynurenine pathway (8). Studies revealed the accumulation of kynurenine pathway metabolites after excessive tryptophan intake in patients with chronic kidney disease undergoing hemodialysis or diabetes mellitus (9, 10) and found clearly that the kynurenine level was parallelly correlated with the degree of renal failure by nephrectomy in rats (11).

The gut microbial metabolites of tryptophan mainly include indole and its derivatives (8). Tryptophan is converted into indole by the intestinal bacteria tryptophanase (TnaA) (12) and travels through the portal vein to the liver. Indoles are further oxidized and sulfated in the liver to form indoxyl sulfate (IS) (13). IS can induce oxidative stress injury in CKD patients, and produce various inflammatory factors and promote vascular endothelial cell damage and kidney damage (14).

It is worth noting that kynurenine and IS are uremic toxins and belong to endogenous ligands of aryl hydrocarbon receptor (15, 16). AhR, a cytoplasmic ligand-activated transcription factor with pleotropic functions, can regulate the expression of cytochrome P450, CyP1A1 and CyP1B1 and is associated with glomerular or tubular damage (17).

So, tryptophan intake may not be the more the better, kynurenine, IS and AhR pathway activation caused by excessive tryptophan metabolism may have adverse effects on the body, especially in patients with chronic kidney disease. In our study we investigated the effects of excessive tryptophan intake on the body and tryptophan metabolism-related AhR pathway in healthy rats and chronic kidney disease rats, to study the adverse effects of excess tryptophan.

## 2. Materials and methods

### 2.1. Experimental animals

Eight-week-old, male Sprague–Dawley (SD) rats were purchased from Southern Medical University Animal Center (Guangzhou, China). Experiments were carried out according to the protocols for animal care and use of laboratory animals, which were approved by the Institutional Animal Care and Research Advisory Committee of Guangdong Provincial Hospital of Chinese Medicine (No.2020027). Until the start of the experiment, the animals were housed in the specific pathogen–free animal breeding room under a temperature-controlled environment on a 12-h light/dark cycle and fed with free water.

### 2.2. Part I experiment-tryptophan dietary intervention in healthy rats

**Figure 1:**
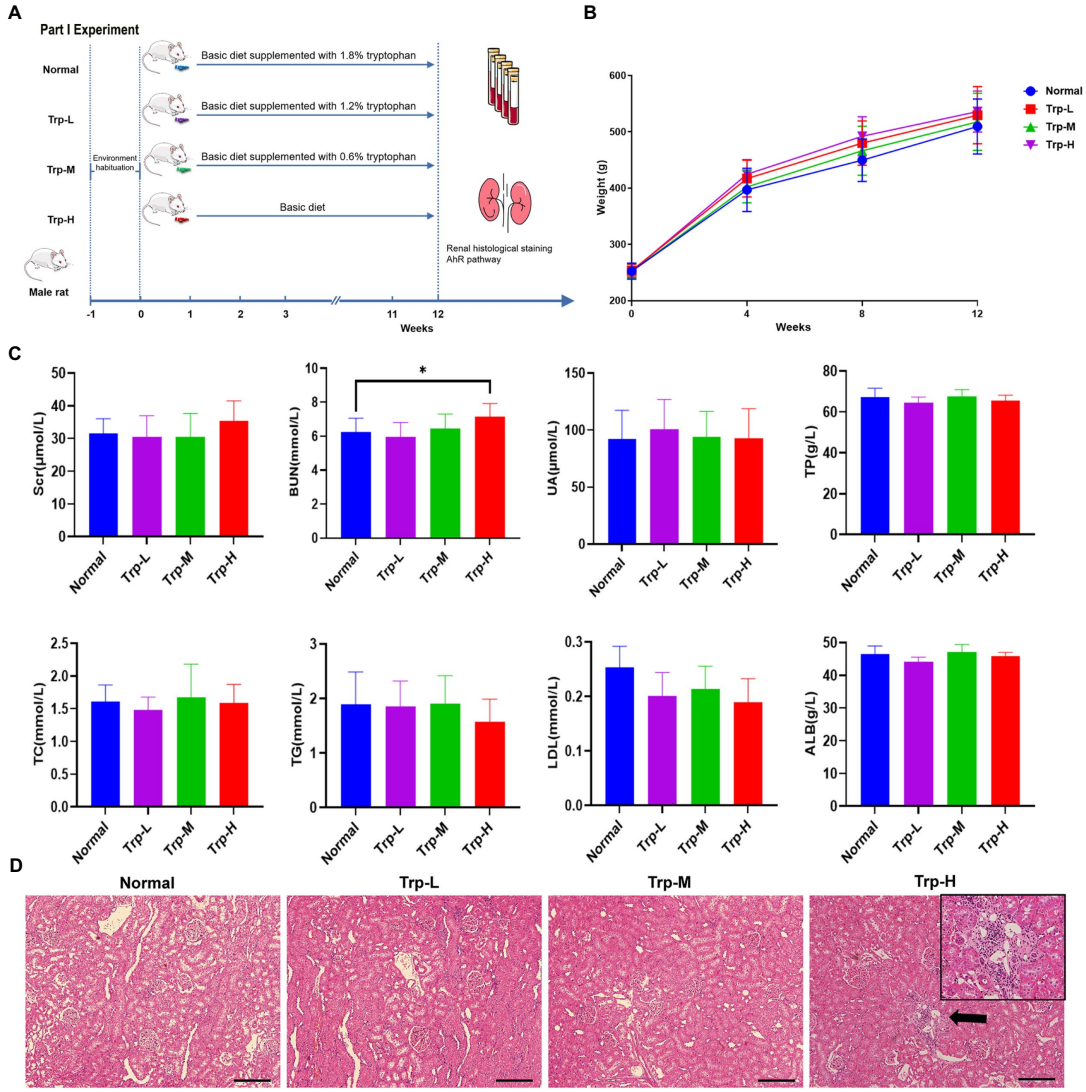
Dietary tryptophan induced kidney injury phenotypes in healthy rats. **(A)** Schedule of Part I experiment. **(B)** Body weight. **(C)** Serum creatinine, blood urea nitrogen, uric acid, total protein, total cholesterol, triglyceride, low density lipoprotein, albumin. **(D)** Hematoxylin–eosin staining of kidney tissue (100×). The data are presented as the mean + standard deviation, n = 10 rats per group (*p < 0.05).

Forty SD rats (healthy) were randomly divided into (1) normal group, (2) 0.6% tryptophan diet group (Trp-L), (3) 1.2% tryptophan diet group (Trp-M), 4) 1.8% tryptophan diet group (Trp-H, *n* = 10, respectively). The tryptophan diet was commissioned to be produced by Guangdong Medical Laboratory Animal Center. The body weights of the rats were recorded at four-week intervals. Tryptophan dietary intervention lasted for 12 weeks (Figure 1A).

### 2.3. Part II experiment-tryptophan intake in adenine-induced chronic kidney disease rats

**Figure 2:**
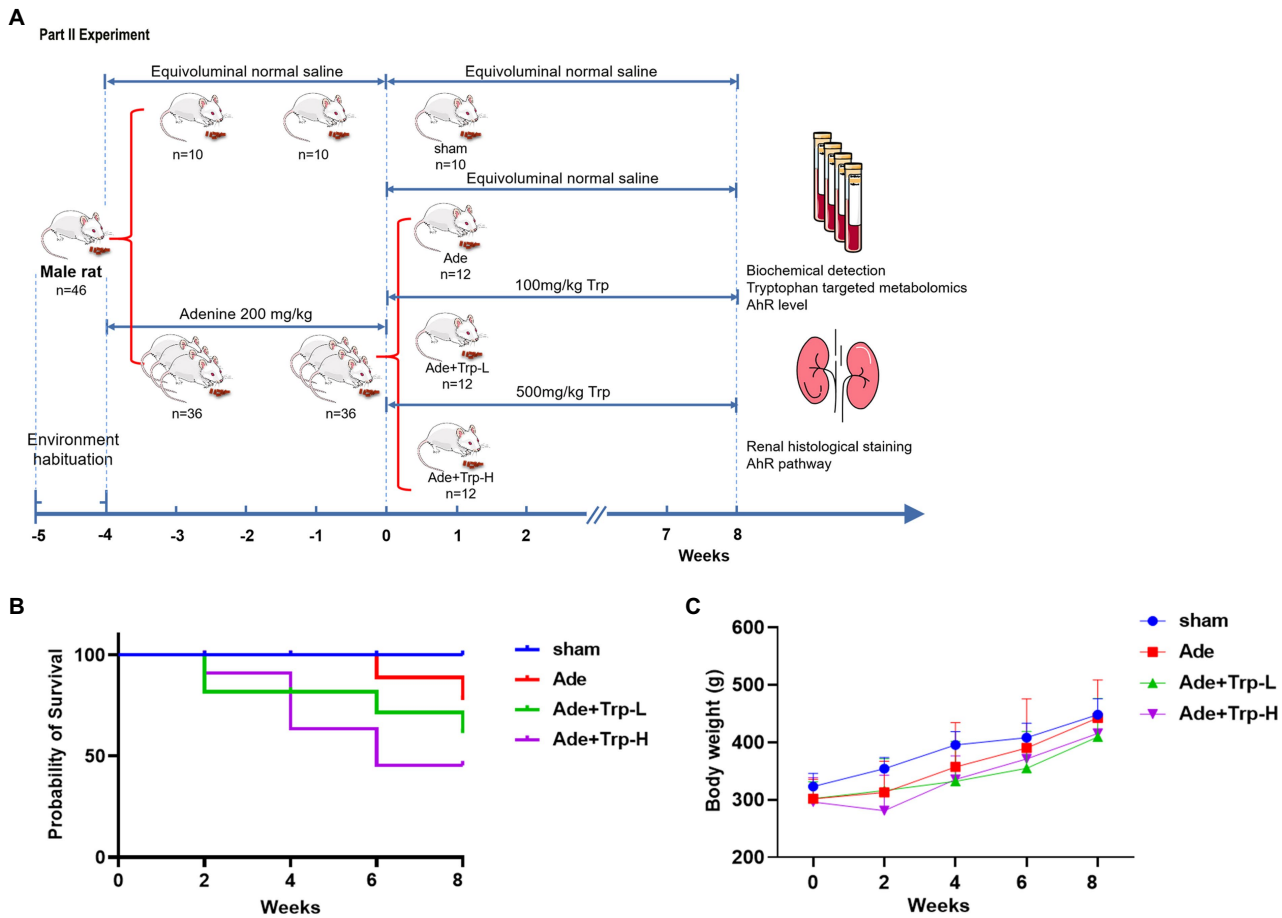
Effect of tryptophan in adenine-induced CKD rats. **(A)** Schedule of Part II experiment. **(B)** Probability of survival. **(C)** Body weight.

Forty-six SD rats were randomly assigned into the sham group (*n* = 10) and the CKD Group (*n* = 36). The CKD group rats was induced by intra-gastric gavage with adenine (Sigma-Aldrich, St Louis, MO, United States) at 200 mg/kg for 4 weeks (18). Then the CKD rats were divided randomly into (1) Adenine Group (Ade), (2) Tryptophan low dose group (Ade + Trp-L) and (3) Tryptophan high dose group (Ade + Trp-H, *n* = 12, respectively). Tryptophan administration rats were given solution of tryptophan at a dose of 100 mg/kg (Ade + Trp-L) and 500 mg/kg (Ade + Trp-H) by intragastric administration once per day for 8 weeks. Sham Group and Adenine Group were administrated with the same volume of normal saline (Figure 2A).

### 2.4. End of experimental intervention

At the end point of the experiment, rats were given an intraperitoneal injection of 2.0% pentobarbital sodium (30 mg/kg body weight) for anesthetization. When rats became unconscious, they were euthanized using cervical dislocation. The abdominal aortic blood and kidney tissues were collected. Blood samples were centrifuged, and all serum samples and part of kidney tissues were stored in −80°C refrigerator, and the remaining renal tissue was fixed with 4% paraformaldehyde for further pathological observation.

### 2.5. Biochemical analysis

Serum creatinine (Scr), blood urea nitrogen (BUN), albumin (ALB), uric acid (UA), total protein (TP), total cholesterol (TC), triglyceride (TG) and low-density lipoprotein (LDL) were measured by Cobas C702 automatic analyzers (Roche, Basel, Switzerland). Proteinuria was measured using a bicinchoninic acid protein detection kit (Thermo Fisher Scientific, Waltham, MA, United States).

### 2.6. Histological analysis

The rat kidney tissues were fixed with 4% paraformaldehyde and then put into dehydrator for dehydration after pruning. After paraffin embedding, the slices were cut into 3 μm thickness and stained with H&E staining (Leagene Biotechnology, Beijing, China) or periodic acid–Schiff (PAS) staining (Nanjing Jiancheng, Jiangsu, China).

### 2.7. Tryptophan-targeted UHPLC-MRM-MS analysis of tryptophan dietary intervention rats

The tryptophan-targeted UHPLC-MRM-MS analysis of rat serum (100 μl) was performed by Shanghai Applied Protein Technology (Shanghai, China).

Sample solution preparation: Standards were purchased from Sigma-Aldrich and Steraloids. Samples were stored at −80°C, and then slowly thawed at 4°C. 100uL of samples were taken from each one, and 400uL of precooled acetonitrile-methanol solution (1:1, v/v) was added, followed by a vortex for 60s. Proteins were precipitated at −20°C for 1 h, centrifuged at 14000 g for 20 min, and the supernatant was freeze-dried, and the samples were stored at −80°C.

UHPLC-MRM-MS Analysis: The UHPLC separation was performed using the UPLC system (Agilent 1,290 Infinity UHPLC) equipped with C-18 column (Waters, CSH C18 1.7 μm, 2.1 mm × 100 mm column). Mobile phase A was acetonitrile, and mobile phase B was water (20 mM ammonium formate buffer as additive to regulate PH to 3.7). The column temperature was set at 50°C and the flow rate was constant at 0.4 ml/min. Parameters of gradient elution mode were as follows: 0-2 min, 15% B; 2–9 min, 15–98% B; 9–11 min, 98% B; 11–11.5 min, 98–15% B; 11.5–14 min, 15% B. The injection volume was 3 μl. The 5,500 QTRAP (AB SCIEX) was performed for assay development. The electron Spray Ionization (ESI) positive source conditions were as follows: Source temperature = 550°C; ion Source Gas1 (Gas1) =55 psi; Ion Source Gas2 (Gas2) =55 psi; Curtain gas (CUR) = 40 psi; ionSapary Voltage Floating (ISVF) = 4,500 V. The atmospheric pressure chemical ionization (APCI) source conditions were as follows: source temperature = 550°C; Gas1 = 55 psi; Gas2 = 55 psi; CUR = 40 psi; ISVF = +5,500 V. MRM method was used to acquire mass spectrometry quantitative data.

### 2.8. Enzyme-linked immunosorbent assay

Serum kynurenic acid and AhR level was measured by enzyme-linked immunosorbent assay (ELISA), referring to the operating’s instructions. Kynurenic acid ELISA kit was obtained from ZCIBIO (Shanghai, China) and AhR ELISA kit was purchased from Cloud-Clone Corp (CCC, TX, United States).

### 2.9. Western blotting analysis

The kidney protein samples were separated by SDS-PAGE electrophoresis and electroblotted onto the nitrocellulose membranes. The membranes were blocked with QuickBlock™ Blocking Buffer for western blot (Beyotime, China) and first incubated overnight at 4°C with primary antibody against AhR (1:500, Santa Cruz, TX, United States), CYP1A1 (1:500; Santa Cruz), CYP1B1 (1:2000, Abcam, United Kingdom), or β-actin (1:8000, Sigma, St. Louis, MO, United States) overnight at 4°C. After being thoroughly washed three times with TBS-T solution, and then incubated with HRP-conjugated secondary antibody. Immunoreactive bands were visualized using chemiluminescent HRP substrate (Millipore, MA, United States) and exposure to the Bio-Rad ChemiDoc XRS^+^ gel imaging system (Hercules, CA, United States).

### 2.10. Statistics analysis

The data were analyzed using SPSS 17.0 software (Chicago, IL, United States). The results are expressed as the mean ± standard deviation (SD). Differences between groups were verified by t-test or one-way ANOVA analysis. A *p* value of <0.05 was considered to indicate a statistically significant result (*) and a p value of <0.01 a highly statistically significant result (**).

## 3. Result

### 3.1. Excessive dietary tryptophan induces kidney injury phenotypes in healthy rats

In part I experiment, we firstly evaluated the effect of excessive tryptophan diet on kidney in healthy rats. No death occurred in rats fed ordinary diet, low, medium and high tryptophan diet. As shown in Figure 1B, body weight of rats in high tryptophan diet slightly increased, but there was no significant difference between four groups. In terms of biochemical indicators (Figure 1C), serum tests serum creatinine (Scr), uric acid (UA), total protein (TP), albumin (ALB), total cholesterol (TC), triglyceride (TG) and low-density lipoprotein (LDL) showed no statistically difference between groups(*p*>0.05). However, blood urea nitrogen (BUN) in Trp-H group was significantly higher than that in normal group (*p* < 0.05), suggesting that azotemia caused by excessive dietary tryptophan. Correspondingly, H&E staining of renal tissues also showed focal renal tubulointerstitial injury in Trp-H rats, the specific manifestations are focal tubular atrophy, interstitial widening, and inflammatory cell infiltration (Figure 1D).

### 3.2. Excessive dietary tryptophan induces serum tryptophan metabolites accumulation in healthy rat

**Figure 3:**
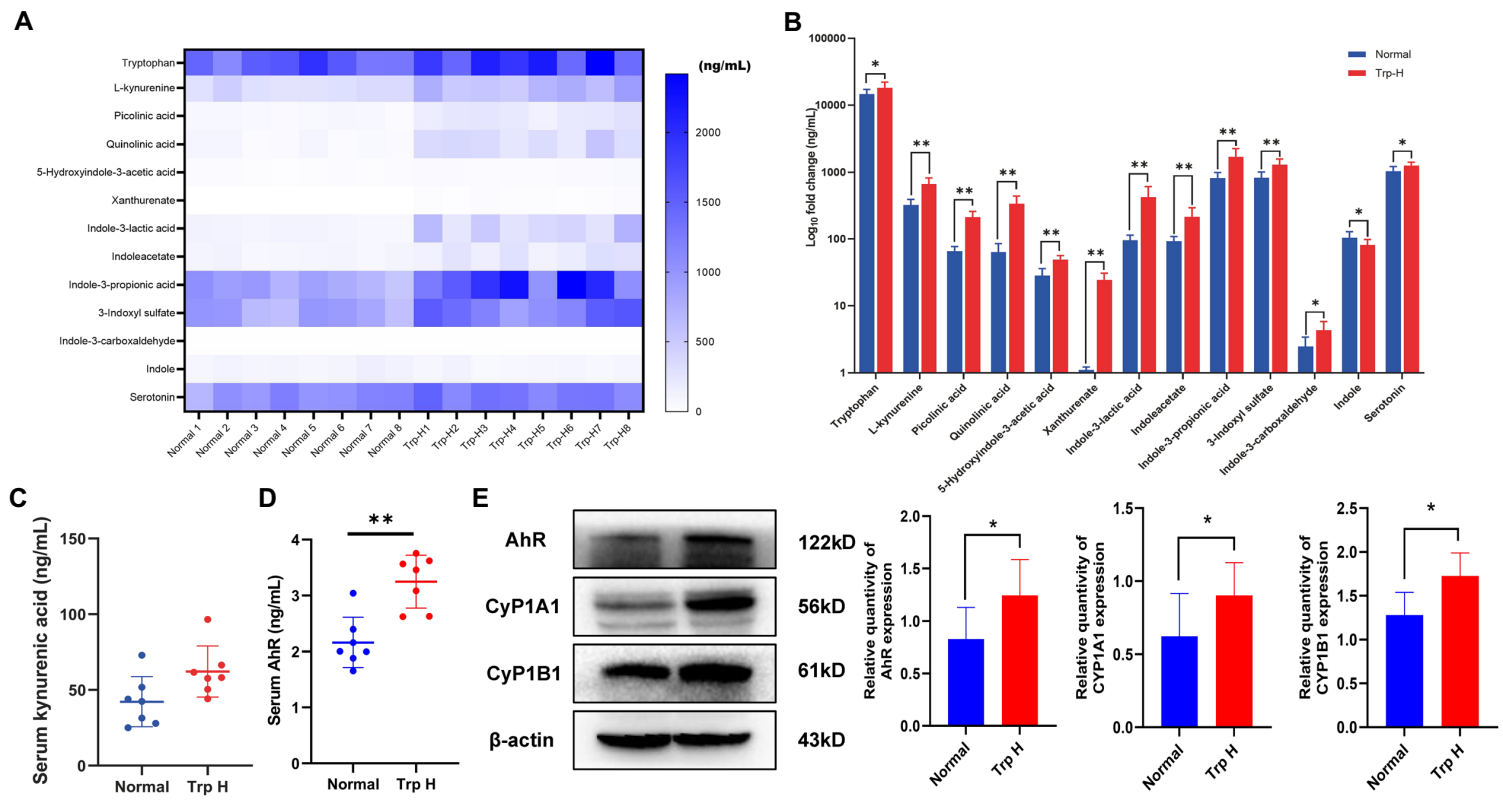
Dietary tryptophan induced tryptophan metabolites accumulation and activation of AhR pathway. **(A,B)** The levels of tryptophan metabolites in serum (*n* = 8). **(C)** Serum kynurenic acid (*n* = 8). **(D)** Serum AhR (*n* = 7). **(E)** Western-blot analysis of AhR, CyP1A1, CyP1B1 expression in kidney tissue of rat with or without tryptophan diet (*n* = 8). The data are presented as the mean + standard deviation (*p < 0.05, **p < 0.01).

UHPLC-MRM-MS technology was used to analyze tryptophan and tryptophan targeted endogenous differential metabolites in serum of both normal and Trp-H rats. Of these metabolites, we are focusing on kynurenine and indole metabolites, such as L-kynurenine, xanthurenate, picolinic acid, quinolinic acid, 5-hydroxyindole-3-acetic acid (5-HIAA), indoleacetate (IAA), indole-3-lactic acid, indoxyl sulfate, indole-3-carboxaldehyde and indole-3-propionic acid. As expected, it can be found that serum tryptophan, L-kynurenine, xanthurenate, picolinic acid, quinolinic acid, 5-hydroxyindole-3-acetic acid, indoleacetate, indole-3-lactic acid, indoxyl sulfate, indole-3-carboxaldehyde and indole-3-propionic acid levels in high-dosage tryptophan diet were significantly higher than in normal group tryptophan (Figures 3A,B). However, the UHPLC-MRM-MS did not successfully detect the concentration of kynurenic acid. But considering that kynurenine is a poor AhR ligand and kynurenine metabolite, kynurenic acid, is the strongest ligand among kynurenine pathway metabolites (15), serum kynurenic acid levels were further determined by ELISA. Compared with normal group, serum kynurenic acid of Trp-H rats shows an upward trend (Figure 3C). These results collectively indicated a positive correlation between the accumulation of tryptophan metabolites amount of excessive tryptophan intake.

### 3.3. Excessive dietary tryptophan causes overexpression of AhR in serum and kidney tissue

Next, as kynurenine and indole derivative are both endogenous ligands of AhR, the expression of AhR and its related proteins CyP1A1 and CyP1B1 were further observed. The result of ELISA found the level of serum AhR in Trp-H group was increased (Figure 3D). Parallelly, western-blot assay also showed the expressions of AhR significantly increased and marked upregulation of CyP1A1 and CyP1B1 in kidney tissue of high-dose-trp-diet rat (Figure 3E).

### 3.4. Bidirectional effect of tryptophan in adenine-induced CKD rats

**Figure 4:**
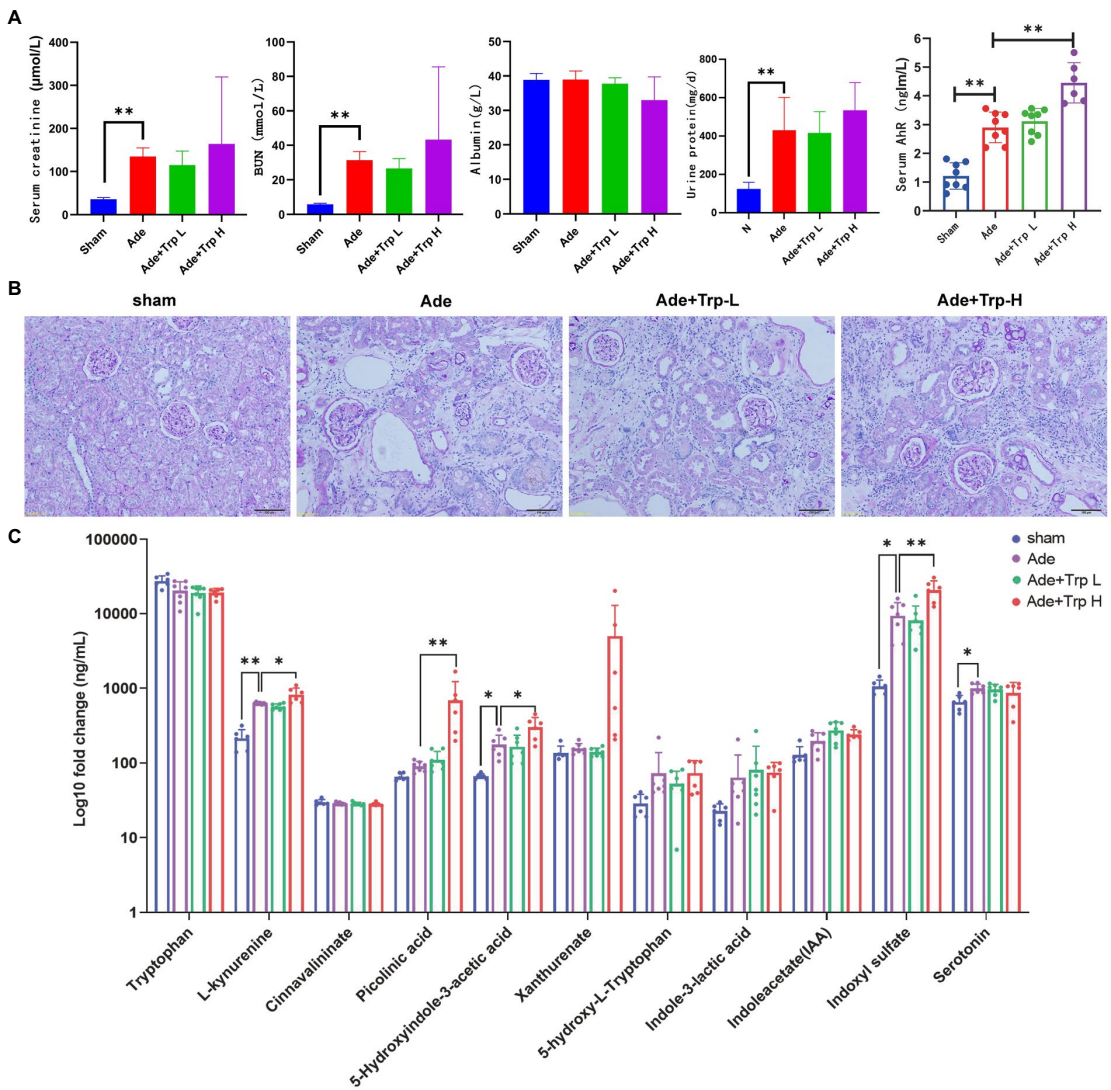
Tryptophan induced Kyn/IS accumulation and AhR pathway activation in CKD rats. **(A)** Serum creatinine, blood urea nitrogen, albumin, urine protein and AhR. **(B)** Periodic acid–Schiff (PAS) staining of kidney tissue (100×). **(C)** The levels of tryptophan metabolites in serum (*n* = 6 in sham and Ade + Trp-H group, *n* = 7 in Ade and Ade + Trp-L group). The data are presented as the means + standard deviation (**p < 0.01).

In part II experiment, we investigated tryptophan intake in adenine-induced chronic kidney disease rats. No death occurred in the sham group during the 8-week observation period. Certain death occurred in adenine group (Ade) and low dose tryptophan group (100 mg/kg, Ade + Trp-L), while adenine rats were administrated with high dose of tryptophan (500 mg/kg, Ade + Trp-H), the death of rats increased significantly from the 4th week (Figure 2B, 50% in 8th week). In terms of body weight, the body weight of the tryptophan intervention group was slightly lower than that of the adenine group (Figure 2C). The levels of serum creatinine (Scr) and blood urea nitrogen (BUN) of adenine rats were significantly higher than those of sham group, and the levels of Scr, BUN and urine protein had a certain downward trend in Ade + Trp-L group rats. The Scr, BUN and urine protein levels were higher and albumin levels were lower in the surviving Ade + Trp-H rats than Ade group rats (Figure 4A).

PAS staining was performed on renal tissue sections to better understand renal lesions. Normal glomeruli and tubule interstitium were observed in sham group. Renal glomeruli in adenine group were basically normal, and the renal interstitial crystals were deposited, tubule atrophy, interstitial widening, and a small amount of cell infiltration with interstitial inflammation. The damage degree of renal tubule interstitial in Ade + Trp-L group was slightly reduced than in adenine group. Compared with adenine group, the damage degree of renal tubule interstitial of the surviving Ade + Trp-H group was aggravated to a certain extent, and the characteristics were significantly increased infiltration of inflammatory cells (Figure 4B).

### 3.5. High dose of tryptophan induced kynurenine/indole metabolites accumulation and AhR activation in CKD rats

Serum tryptophan metabolites in adenine-induced CKD rats were also determined by UHPLC-MRM-MS technology. Serum kynurenine, 5-hydroxyindole-3-acetic acid, indoxyl sulfate and serotonin showed significantly increased and picolinic acid, xanthurenate, 5-hydroxy-L-tryptophan, indole-3-lactic acid, indoleacetate also showed upward trends in the Ade + Trp-H group compared with sham group. In the Ade + Trp-L group, kynurenine, 5-hydroxyindole-3-acetic acid, xanthurenate, 5-hydroxy-L-tryptophan, indoxyl sulfate showed slight declines compared with the Ade group. However, notedly, kynurenine, picolinic acid, 5-hydroxyindole-3-acetic acid and indoxyl sulfate increased significantly in Ade + Trp-H group compared with the Ade group (Figure 4C). Further, compared with sham group, serum AhR of adenine-induced CKD rats were significantly up-regulated. In the Ade + Trp-L group, AhR showed no significant difference compared with adenine group. The serum AhR of Ade + Trp-H group were significantly higher than those of adenine rats (Figure 4A).

## 4. Discussion

In the past decades, studies have found that tryptophan metabolic disorder is associated with inflammatory bowel disease, ulcerative colitis, irritable bowel syndrome (19, 20), metabolic syndrome and related complications such as diabetes, obesity, non-alcoholic fatty liver disease, insulin resistance, atherosclerosis, anxiety, depression and autism (21–23). Dietary tryptophan and its metabolites appear to have a potential role in the treatment of these disease (8).

Tryptophan metabolism pathway can prevent and alleviate the occurrence and development of intestinal inflammation by directly or indirectly regulating the balance between intestinal proinflammatory/anti-inflammatory cytokines, such as interferon-γ (IFN-γ), transforming growth factor-β (TGF-β), tumor necrosis factor (TNF-α), interleukin-10 (IL-10) and interleukin-22 (IL-22), the function of immune cells (DCs, NK cells, B cells, T-regs and Macrophages), intestinal microbial composition and homeostasis (24). Tryptophan metabolism in intestine has become the star of disease treatment. Although tryptophan has important nutritional value and is widely used in medical applications (25), toxicity data for oral management are relatively limited.

Therefore, it is necessary to evaluate its safety. An article proposed a tryptophan-intake upper limit of 4.5 g/d (26), but more clinical and laboratory data are lacking. In order to explore the potential effects of excessive tryptophan on the body, this study systematically studied the effects of excessive intake of tryptophan in healthy and diseased rats. Notedly, a previous study found that healthy women did not experience any adverse effects after excessive tryptophan intake, but abruptly increased urinal tryptophan/kynurenine metabolites at 5 g/d tryptophan intake (27). Unfortunately, blood tryptophan metabolites concentrations were not detected successfully, and the observation period was only 3 weeks in this study. The nephrotoxicity of tryptophan needs further study. In our part I experiment, when healthy rats were fed diets supplemented with excessive L-tryptophan (1.8%) for 3 months, the blood urea nitrogen level of rats was significantly increased, and the kidney showed small focal tubule interstitial injury. The UHPLC-MRM-MS showed the tryptophan metabolites were significantly increased in healthy rats and with an upward trend in adenine-induced CKD rats, especially kynurenine and indoxyl sulfate (IS).

Considering that tryptophan-derived uremic toxins accumulation caused by excessive intake of tryptophan may affect the progression of chronic kidney disease (28), the disease model of this study we chose a chronic kidney disease model. A review of the “renal protective” function of tryptophan hypothesized that dietary tryptophan can maintain renal tubular metabolic health and attenuate long-term profibrotic responses that could lead to chronic kidney disease (29). However, this hypothesis has not been tested on an ischemia–reperfusion mouse treated with or without 400 mg/kg tryptophan-derived nicotinamide (NAM), and increased kidney damage molecule-1 (Kim-1) mRNA expression deserved further discussion (30). More evidence of kidney-injury predisposition was further obtained in our part II experiment. It was found in adenine-induced CKD rats that low dose of tryptophan may had a potential therapeutic effect on adenine induced CKD rats. It was shown that the Scr and BUN levels of rats were decreased to a certain extent, and the kidney pathological injury was alleviated in Ade + Trp-L group. However, after the intervention of Ade + Trp-H, the mortality of rats increased significantly (50% by 8 weeks), the levels of Scr, BUN and urine protein increased, the pathological injury of the kidney was aggravated, and the infiltration of inflammatory cells was enhanced. The serum levels of kynurenine, kynurenine metabolites and indole metabolites were significantly increased in the Ade + Trp-H group.

L-kynurenine, xanthurenic acid, quinolinic acid, indoleacetate (IAA), indole-3-lactic acid, indoxyl sulfate indicated excess L-tryptophan is metabolized through the kynurenine and indole derivatives metabolic pathways both in healthy rats and adenine-induced CKD rats. The increase in Kyn suggests tryptophan 2,3- dioxygenase (TDO)/indoleamine 2,3- dioxygenase (IDO) activation and that in quinolinic acid implies enhanced KP enzymes (31). Unfortunately, we were not able to detect proximal Kyn metabolites 3-hydroxykynurenine (3-HK) and 3-hydroxyanthranilicacid (3-HKK), and the absence of partial results of proximal metabolites limits the explanation of the damage mechanism of kyn pathway. The above results are consistent with the previous findings that the accumulation of serum L-kynurenine metabolites is proportional to the severity of renal failure (11, 32).

Further, tryptophan metabolites including indole, IAA, indole acrylic, and indole-3-aldehyde serve as ligands for aryl hydrocarbon receptor (AhR) to enhance intestinal epithelial barrier function by activating the AhR pathway to up-regulate the expression of genes involved in maintaining the structure and function of intestinal epithelial cells. Tryptophan metabolites play an active role in intestinal immune disorder and intestinal mucosal barrier damage. For chronic kidney disease, our previous literature review also found the dual effect of AhR (33). On the one hand, the intestinal flora is disturbed and the intestinal mucosal barrier function is impaired, so tryptophan supplementation may play a positive role by regulating the intestinal AhR (34). On the other hand, Kyn and IS are uremic toxins, and excessive accumulation of them in serum or organs will activate circulation and tissue AhR (35). The balance between these opposites of AhR will determine the level of harm.

Taken above together, excess L-tryptophan is metabolized through the kynurenine and indole derivatives metabolic pathways, and that kynurenine and indole derivatives, especially indoxyl sulfate, have been identified as AhR ligands (36, 37). AhR is a transcription factor widely expressed in immune cells, activation of which alters innate and adaptive immune responses in ligand-dependent ways. A previous study had confirmed that endogenous tryptophan metabolites can bind and activate AhR *in vitro* and *in vivo*. Meanwhile AhR has also been shown to mediate inflammation and cardiovascular disease in CKD patients (38). Based on current research, most of the tryptophan metabolites belong to uremic toxins, and cannot be efficiently eliminated by common dialysis modalities (39), long-term accumulation of which in the body will accelerate renal failure. Renal AhR expression was positively correlated with the severity of CKD in a nephrectomy male rat experiment (40) and found that AhR can induce renal pro-inflammatory phenotypes (6), podocyte damage (41), and glomerular damage (42).

Correspondingly, the expression of AhR in serum or kidney tissue were increased significantly after feeding 1.8% tryptophan in healthy rats for 3 months. The expressions of CyP1A1 and CyP1B1 were also increased in kidney tissue. The serum AhR levels of adenine rats were also significantly increased after ingesting high dose of tryptophan. Therefore, combined with those results collected above, we inferred that excessive tryptophan intake does induce renal injury in healthy rats and it also aggravated the kidney damage and increased the mortality of CKD rats, which may be induced by the Trp-Kyn/IS-AhR pathway.

## 5. Conclusion

Tryptophan metabolism affects the physiological and pathological effects of the host. There are three ways of tryptophan metabolism, including kynurenine, serotonin and indole derivatives, which play an important role in the occurrence and development of diseases. The biological effects of tryptophan metabolite and the changes in disease state suggest that tryptophan metabolite may be a therapeutic target.

So far, tryptophan metabolism and its related biochemical pathways still need to be further described, and the complex factors of this task include a wide variety of tryptophan metabolites and complex intestinal microbial metabolism, which requires further research to improve the target and intervention measures. Prior to this, intervention with excessive of tryptophan had potential risks, especially for chronic kidney disease. Kynurenine and indole pathways of tryptophan metabolism play an important role in the development of healthy and chronic kidney disease. The intake of tryptophan is not the more the better. However, due to the limitation of this study, it is not clear to determine the threshold of damage caused by excessive tryptophan, and whether there is a difference between short-term and long-term supplementation of this amino acid. The mechanisms and targets of kynurenine and indole derivatives metabolic pathways still need to be further studied.

## Data availability statement

The original contributions presented in the study are included in the article/supplementary material, further inquiries can be directed to the corresponding authors.

## Ethics statement

The animal study was reviewed and approved by The Institutional Animal Care and Research Advisory Committee of Guangdong Provincial Hospital of Chinese Medicine.

## Author contributions

ZL and WM: study concept and design. DH, JL, and ZL: animal handling and biochemical analysis. WY and LH: histological analysis. DH, JL, and CL: ELISA and western-blot assay. DH, JL, WM, and ZL: drafting of the manuscript. WM and ZL: critical revision of the manuscript for important intellectual content and obtained funding. All authors have read and approved the manuscript.

## Funding

This project was supported by the Specific Fund of State Key Laboratory of Dampness Syndrome of Chinese Medicine (SZ2021ZZ1004).

## Conflict of interest

The authors declare that the research was conducted in the absence of any commercial or financial relationships that could be construed as a potential conflict of interest.

## Publisher’s note

All claims expressed in this article are solely those of the authors and do not necessarily represent those of their affiliated organizations, or those of the publisher, the editors and the reviewers. Any product that may be evaluated in this article, or claim that may be made by its manufacturer, is not guaranteed or endorsed by the publisher.

## Abbreviations

5-HIAA: 5-hydroxyindole-3-acetic acid
3-HK: 3-hydroxykynurenine
3-HKK: 3-hydroxyanthranilicacid
Ade: adenine
AhR: aryl hydrocarbon receptor
ALB: albumin
APCI: atmospheric pressure chemical ionization
BUN: blood urea nitrogen
CUR: Curtain gas
ECL: enhanced chemiluminescence assay
ESI: Electron Spray Ionization
Gas1: ion Source Gas1
Gas2: Ion Source Gas2
IS: indoxyl sulfate
H&E: hematoxylin–eosin
IAA: indoleacetic acid
IDO: indoleamine 2,3- dioxygenase
IFN-γ: interferon-γ
IL-10: interleukin-10
IL-22: interleukin-22
ISVF: ionSapary Voltage Floating
Kim-1: kidney damage molecule-1
KP: kynurenine pathway
Kyn: kynurenine
LDL: low-density lipoprotein
NAD+: nicotinamide adenine dinucleotide
NAM: nicotinamide
NC: Nitrocellulose
PAS: periodic acid–Schiff
Scr: Serum creatinine
SPF: specific pathogen free
TC: total cholesterol
TDO: tryptophan 2,3- dioxygenase
TG: triglyceride
TGF-β: transforming growth factor-β
TnaA: tryptophanase
TNF-α: tumor necrosis factor
TP: total protein
Trp: tryptophan
UA: uric acid
UHPLC-MRM-MS: ultra-high-performance liquid chromatography coupled with multiple reaction monitoring mass spectrometry

## References

[1] Nikolaus S Schulte B Al-Massad N Thieme F Schulte DM Bethge J. Increased tryptophan metabolism is associated with activity of inflammatory bowel diseases. Gastroenterology. (2017) 153:1504–1516.e2. doi: 10.1053/j.gastro.2017.08.028, PMID: 28827067

[2] Zamoscik V Schmidt SNL Bravo R Ugartemendia L Plieger T Rodriguez AB. Tryptophan-enriched diet or 5-hydroxytryptophan supplementation given in a randomized controlled trial impacts social cognition on a neural and behavioral level. Sci Rep. (2021) 11:21637. doi: 10.1038/s41598-021-01164-y, PMID: 34737364 PMC8568973

[3] Meier TB Savitz J. The kynurenine pathway in traumatic brain injury: implications for psychiatric outcomes. Biol Psychiatry. (2022) 91:449–58. doi: 10.1016/j.biopsych.2021.05.021, PMID: 34266671 PMC8630076

[4] Platten M Nollen EAA Rohrig UF Fallarino F Opitz CA. Tryptophan metabolism as a common therapeutic target in cancer, neurodegeneration and beyond. Nat Rev Drug Discov. (2019) 18:379–401. doi: 10.1038/s41573-019-0016-5, PMID: 30760888

[5] Clarke G Grenham S Scully P Fitzgerald P Moloney RD Shanahan F. The microbiome-gut-brain axis during early life regulates the hippocampal serotonergic system in a sex-dependent manner. Mol Psychiatry. (2013) 18:666–73. doi: 10.1038/mp.2012.77, PMID: 22688187

[6] Tao S Guo F Ren Q Liu J Wei T Li L. Activation of aryl hydrocarbon receptor by 6-formylindolo[3,2-b]carbazole alleviated acute kidney injury by repressing inflammation and apoptosis. J Cell Mol Med. (2021) 25:1035–47. doi: 10.1111/jcmm.16168, PMID: 33280241 PMC7812300

[7] Zhang J Zhu S Ma N Johnston LJ Wu C Ma X. Metabolites of microbiota response to tryptophan and intestinal mucosal immunity: a therapeutic target to control intestinal inflammation. Med Res Rev. (2021) 41:1061–88. doi: 10.1002/med.21752, PMID: 33174230

[8] Agus A Planchais J Sokol H. Gut microbiota regulation of tryptophan metabolism in health and disease. Cell Host Microbe. (2018) 23:716–24. doi: 10.1016/j.chom.2018.05.003, PMID: 29902437

[9] Matsuoka K Kato K Takao T Ogawa M Ishii Y Shimizu F. Concentrations of various tryptophan metabolites are higher in patients with diabetes mellitus than in healthy aged male adults. Diabetol Int. (2017) 8:69–75. doi: 10.1007/s13340-016-0282-y, PMID: 30603309 PMC6224928

[10] Post A Huberts M Poppe E Faassen MV Kema IP Vogels S. Tryptophan intake and tryptophan losses in hemodialysis patients: a balance study. Nutrients. (2019) 11:2851. doi: 10.3390/nu11122851, PMID: 31766383 PMC6950375

[11] Saito K Fujigaki S Heyes MP Shibata K Takemura M Fujii H. Mechanism of increases in L-kynurenine and quinolinic acid in renal insufficiency. Am J Physiol Renal Physiol. (2000) 279:F565–72. doi: 10.1152/ajprenal.2000.279.3.F565, PMID: 10966936

[12] Sasaki-Imamura T Yoshida Y Suwabe K Yoshimura F Kato H. Molecular basis of indole production catalyzed by tryptophanase in the genus *Prevotella*. FEMS Microbiol Lett. (2011) 322:51–9. doi: 10.1111/j.1574-6968.2011.02329.x, PMID: 21658104

[13] Devlin AS Marcobal A Dodd D Nayfach S Plummer N Meyer T. Modulation of a circulating uremic solute via rational genetic manipulation of the gut microbiota. Cell Host Microbe. (2016) 20:709–15. doi: 10.1016/j.chom.2016.10.021, PMID: 27916477 PMC5159218

[14] Graboski AL Redinbo MR. Gut-derived protein-bound uremic toxins. Toxins (Basel). (2020) 12:590. doi: 10.3390/toxins12090590, PMID: 32932981 PMC7551879

[15] DiNatale BC Murray IA Schroeder JC Flaveny CA Lahoti TS Laurenzana EM. Kynurenic acid is a potent endogenous aryl hydrocarbon receptor ligand that synergistically induces interleukin-6 in the presence of inflammatory signaling. Toxicol Sci. (2010) 115:89–97. doi: 10.1093/toxsci/kfq024, PMID: 20106948 PMC2855350

[16] Opitz CA Litzenburger UM Sahm F Ott M Tritschler I Trump S. An endogenous tumour-promoting ligand of the human aryl hydrocarbon receptor. Nature. (2011) 478:197–203. doi: 10.1038/nature10491, PMID: 21976023

[17] Curran CS Kopp JB. Aryl hydrocarbon receptor mechanisms affecting chronic kidney disease. Front Pharmacol. (2022) 13:782199. doi: 10.3389/fphar.2022.782199, PMID: 35237156 PMC8882872

[18] Thakur R Sharma A Lingaraju MC Begum J Kumar D Mathesh K. Ameliorative effect of ursolic acid on renal fibrosis in adenine-induced chronic kidney disease in rats. Biomed Pharmacother. (2018) 101:972–80. doi: 10.1016/j.biopha.2018.02.143, PMID: 29635907

[19] Lavelle A Sokol H. Gut microbiota-derived metabolites as key actors in inflammatory bowel disease. Nat Rev Gastroenterol Hepatol. (2020) 17:223–37. doi: 10.1038/s41575-019-0258-z, PMID: 32076145

[20] Tuchaai E Endres V Jones B Shankar S Klemashevich C Sun Y. Deletion of ghrelin alters tryptophan metabolism and exacerbates experimental ulcerative colitis in aged mice. Exp Biol Med (Maywood). (2022) 247:1558–69. doi: 10.1177/15353702221110647, PMID: 35833540 PMC9554169

[21] Peirce JM Alviña K. The role of inflammation and the gut microbiome in depression and anxiety. J Neurosci Res. (2019) 97:1223–41. doi: 10.1002/jnr.24476, PMID: 31144383

[22] Ralli T Saifi Z Tyagi N Vidyadhari A Aeri V Kohli K. Deciphering the role of gut metabolites in non-alcoholic fatty liver disease. Crit Rev Microbiol. (2022) 1:1–19. doi: 10.1080/1040841x.2022.2142091, PMID: 36394607

[23] Taleb S. Tryptophan dietary impacts gut barrier and metabolic diseases. Front Immunol. (2019) 10:2113. doi: 10.3389/fimmu.2019.02113, PMID: 31552046 PMC6746884

[24] Li X Zhang ZH Zabed HM Yun J Zhang G Qi X. An insight into the roles of dietary tryptophan and its metabolites in intestinal inflammation and inflammatory bowel disease. Mol Nutr Food Res. (2021) 65:e2000461. doi: 10.1002/mnfr.202000461, PMID: 33216452

[25] Comai S Bertazzo A Brughera M Crotti S. Tryptophan in health and disease. Adv Clin Chem. (2020) 95:165–218. doi: 10.1016/bs.acc.2019.08.005 32122523

[26] Cynober L Bier DM Kadowaki M Morris SM Jr Elango R Smriga M. Proposals for upper limits of safe intake for arginine and tryptophan in young adults and an upper limit of safe intake for leucine in the elderly. J Nutr. (2016) 146:2652s–4s. doi: 10.3945/jn.115.228478, PMID: 27934658

[27] Hiratsuka C Fukuwatari T Sano M Saito K Sasaki S Shibata K. Supplementing healthy women with up to 5.0 g/d of L-tryptophan has no adverse effects. J Nutr. (2013) 143:859–66. doi: 10.3945/jn.112.173823, PMID: 23616514

[28] Kamiński TW Pawlak K Karbowska M Myśliwiec M Pawlak D. Indoxyl sulfate - the uremic toxin linking hemostatic system disturbances with the prevalence of cardiovascular disease in patients with chronic kidney disease. BMC Nephrol. (2017) 18:35. doi: 10.1186/s12882-017-0457-1, PMID: 28122514 PMC5267373

[29] Ralto KM Rhee EP Parikh SM. NAD(+) homeostasis in renal health and disease. Nat Rev Nephrol. (2020) 16:99–111. doi: 10.1038/s41581-019-0216-6, PMID: 31673160 PMC7223841

[30] Piedrafita A Balayssac S Mayeur N Gazut S Grossac J Buleon M. The tryptophan pathway and nicotinamide supplementation in ischaemic acute kidney injury. Clin Kidney J. (2021) 14:2490–6. doi: 10.1093/ckj/sfab050, PMID: 34950461 PMC8690092

[31] Cervenka I Agudelo LZ Ruas JL. Kynurenines: Tryptophan's metabolites in exercise, inflammation, and mental health. Science. (2017) 357:eaaf9794. doi: 10.1126/science.aaf9794, PMID: 28751584

[32] Pawlak D Tankiewicz A Matys T Buczko W. Peripheral distribution of kynurenine metabolites and activity of kynurenine pathway enzymes in renal failure. J Physiol Pharmacol. (2003) 54:175–89. 12832720

[33] Mo Y Lu Z Wang L Ji C Zou C Liu X. The aryl hydrocarbon receptor in chronic kidney disease: friend or foe? Front Cell Dev Biol. (2020) 8:589752. doi: 10.3389/fcell.2020.589752, PMID: 33415104 PMC7784643

[34] Hsu CN Lin IC Yu HR Huang LT Tiao MM Tain YL. Maternal tryptophan supplementation protects adult rat offspring against hypertension programmed by maternal chronic kidney disease: implication of tryptophan-metabolizing microbiome and aryl hydrocarbon receptor. Int J Mol Sci. (2020) 21:4552. doi: 10.3390/ijms21124552, PMID: 32604820 PMC7349830

[35] Liu JR Miao H Deng DQ Vaziri ND Li P Zhao YY. Gut microbiota-derived tryptophan metabolism mediates renal fibrosis by aryl hydrocarbon receptor signaling activation. Cell Mol Life Sci. (2021) 78:909–22. doi: 10.1007/s00018-020-03645-1, PMID: 32965514 PMC11073292

[36] Campesato LF Budhu S Tchaicha J Weng CH Gigoux M Cohen IJ. Blockade of the AHR restricts a Treg-macrophage suppressive axis induced by L-kynurenine. Nat Commun. (2020) 11:4011. doi: 10.1038/s41467-020-17750-z, PMID: 32782249 PMC7419300

[37] Szelest M Walczak K Plech T. A new insight into the potential role of tryptophan-derived AhR ligands in skin physiological and pathological processes. Int J Mol Sci. (2021) 22:1104. doi: 10.3390/ijms22031104, PMID: 33499346 PMC7865493

[38] Brito JS Borges NA Esgalhado M Magliano DC Soulage CO Mafra D. Aryl hydrocarbon receptor activation in chronic kidney disease: role of uremic toxins. Nephron. (2017) 137:1–7. doi: 10.1159/000476074, PMID: 28490014

[39] Zakrocka I Zaluska W. Kynurenine pathway in kidney diseases. Pharmacol Rep. (2022) 74:27–39. doi: 10.1007/s43440-021-00329-w, PMID: 34617264 PMC8786771

[40] Shyu JF Liu WC Zheng CM Fang TC Hou YC Chang CT. Toxic effects of Indoxyl sulfate on Osteoclastogenesis and Osteoblastogenesis. Int J Mol Sci. (2021) 22:11265. doi: 10.3390/ijms222011265, PMID: 34681927 PMC8538618

[41] Falahatpisheh MH Ramos KS. Ligand-activated Ahr signaling leads to disruption of nephrogenesis and altered Wilms' tumor suppressor mRNA splicing. Oncogene. (2003) 22:2160–71. doi: 10.1038/sj.onc.1206238, PMID: 12687018

[42] Li MZ Zhao Y Wang HR Talukder M Li JL. Lycopene preventing DEHP-induced renal cell damage is targeted by aryl hydrocarbon receptor. J Agric Food Chem. (2021) 69:12853–61. doi: 10.1021/acs.jafc.1c05250, PMID: 34670089

